# Response surface methodology for process optimization in livestock wastewater treatment: A review

**DOI:** 10.1016/j.heliyon.2024.e30326

**Published:** 2024-04-24

**Authors:** Arif Reza, Lide Chen, Xinwei Mao

**Affiliations:** a Department of Soil and Water Systems, Twin Falls Research and Extension Center, University of Idaho, 315 Falls Avenue, Twin Falls, ID, 83303-1827, USA; b New York State Center for Clean Water Technology, Stony Brook University, Stony Brook, 11794-5000, USA; c School of Marine and Atmospheric Sciences, Stony Brook University, Stony Brook, NY, 11794-5000, USA; d Department of Civil Engineering, Stony Brook University, Stony Brook, NY, 11794-4424, USA

**Keywords:** Response surface methodology, Livestock wastewater treatment, Experimental design, Process optimization, Pollutant removal, Sustainability

## Abstract

With increasing demand for meat and dairy products, the volume of wastewater generated from the livestock industry has become a significant environmental concern. The treatment of livestock wastewater (LWW) is a challenging process that involves removing nutrients, organic matter, pathogens, and other pollutants from livestock manure and urine. In response to this challenge, researchers have developed and investigated different biological, physical, and chemical treatment technologies that perform better upon optimization. Optimization of LWW handling processes can help improve the efficacy and sustainability of treatment systems as well as minimize environmental impacts and associated costs. Response surface methodology (RSM) as an optimization approach can effectively optimize operational parameters that affect process performance. This review article summarizes the main steps of RSM, recent applications of RSM in LWW treatment, highlights the advantages and limitations of this technique, and provides recommendations for future research and practice, including its cost-effectiveness, accuracy, and ability to improve treatment efficiency.

## Highlights

• Holistic overview of the RSM application in LWW treatment was presented for the first time.
• RSM strategy and its significance in LWW treatment were explained in detail.
• Parameters that critically influence the treatment process performance were reported.
• Potential enhancements and future perspectives regarding RSM application in LWW treatment were discussed.

## Graphical abstract

**Figure:**
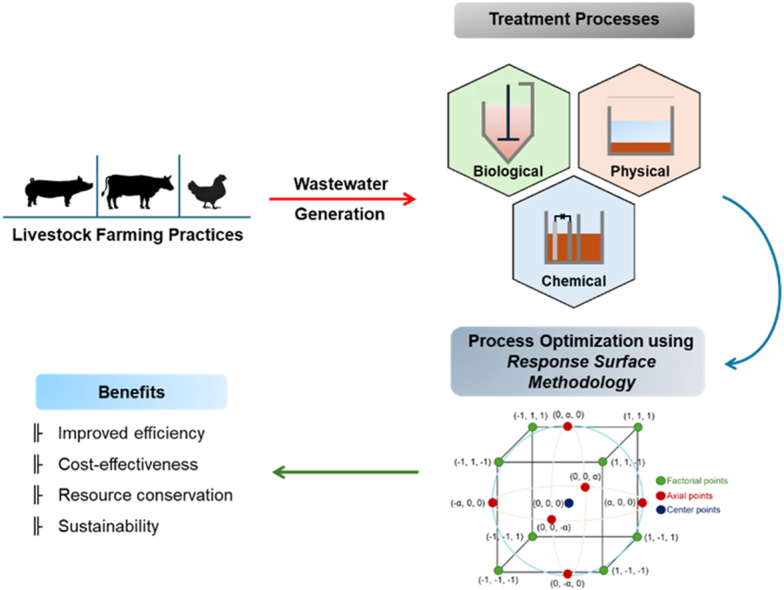

## 1 Introduction

The livestock industry is an important aspect of the agricultural farming system, providing a major source of protein and livelihood for millions of people worldwide. However, it is also associated with generating large volumes of wastewater [1,2]. Livestock wastewater (LWW), also known as animal manure or farm effluent, refers to the liquid waste generated from animal production facilities, such as dairy farms, swine farms, and poultry farms. It contains a complex mixture of organic and inorganic pollutants, including nutrients (such as nitrogen and phosphorus), pathogens, heavy metals, antibiotics, hormones, and other contaminants [3]. Discharging untreated or improperly treated LWW can harm the environment, including water pollution, soil degradation, and negative impacts on aquatic ecosystems as well as lead to numerous human health concerns [[4], [5], [6], [7]]. The increasing intensification and expansion of livestock production have further exaggerated the overall environmental and social challenges. Therefore, the effective treatment of LWW is crucial to minimize its environmental impact and ensure compliance with regulatory requirements [8].

Process optimization plays an important role in designing and developing LWW treatment technologies as it ensures efficient use of resources, improves treatment efficiency, reduces environmental impact, and meets effluent discharge standards [[9], [10], [11], [12], [13]]. Furthermore, Den and Huang [14] emphasized the significance of process optimization in upscaling wastewater treatment processes. Considering the limitations of conventional one-factor-at-a-time (OFAT) optimization approaches, response surface methodology (RSM) as a more accurate and robust optimization technique was first introduced in the middle of 20th century. Response Surface Methodology has several advantages over traditional OFAT optimization approaches, including multiple response optimization, reduced experimentation, improved understanding of the process interactions, and enhanced prediction accuracy [15,16]. Response Surface Methodology, as an optimization approach, therefore, not only helps to overcome the limitations of the OFAT optimization process but also shows superior performance when it is applied in different branches of science and engineering [17,18]. Response Surface Methodology combines mathematical and statistical approaches used to design experiments, analyze data, and develop predictive models for process optimization [19]. This particular optimization approach is based on the concept of response surfaces [20], which are mathematical models that describe the relationship between the input variables (also regarded as independent parameters or factors) and output variables (also interpreted as response or dependent parameters) of a process [21]. Furthermore, it allows for the efficient and systematic exploration of the input variable space to identify optimal process conditions that can result in improved performance and reduced variability [22]. However, RSM also has some limitations, such as the exclusion of non-controllable input variables, assumption of linearity, the need for adequate data for model fitting, and the inability to integrate non-controllable parameters and potential challenges in optimizing complex and nonlinear systems [[23], [24], [25], [26], [27]]. In addition, the accuracy and reliability of RSM models depend on the quality of the experimental observations, and the presence of noise or variability in the data may affect the accuracy of the model predictions [28]. Moreover, the experimental design and statistical analysis in RSM may become complex when dealing with five or more input factors, requiring careful consideration and expertise in model development and analysis [29].

Despite the limitations, RSM has successfully been applied to various LWW treatment processes to ensure efficient and effective removal of pollutants from livestock waste streams. For example, RSM has been used to optimize physical treatment processes such as air-recirculated stripping [30], aqueous ammonia soaking pretreatment [31], microwave-assisted digestion [32], microwave-assisted thermal stripping [33], etc. In the case of chemical processes, RSM has been used to optimize important parameters for electrocoagulation, precipitation, adsorption, pyrolysis, and Fenton processes to accomplish maximum removal of pollutants [13,[34], [35], [36], [37]]. In biological processes, RSM has been applied to optimize the operating conditions of anaerobic digestion, co-digestion, fermentation, microalgal treatment, etc. to maximize pollutant removal and biogas production [[38], [39], [40], [41], [42]]. These studies have demonstrated the effectiveness and versatility of RSM in LWW treatment process optimization and control. RSM, in general, provides a systematic approach for designing experiments, collecting data, and developing mathematical models that can help in understanding the interactions among different process parameters and their effects on treatment performance [33].

Up till now, most review articles regarding RSM application for optimization have focused on fields like analytical chemistry [43], natural product research [44], engineering [45], aerodynamic analyses [46], food industry [47], product design [19], biofuel production [48], metabolomics-related studies [49], and pharmaceutical industry [50]. Although the application of RSM in the LWW treatment sector is well documented, an informed and comprehensive review regarding its applicability in this domain is still unavailable. There is also a lack of holistic guidelines for RSM application in the LWW treatment field. Furthermore, research gaps regarding RSM application in LWW treatment persist in several key areas. For instance, the impact of seasonal and environmental factors including pH, temperature, aeration, mixing intensity, C/N ration, residence time, etc. on RSM-based optimization approaches remains understudied which impedes the real-world application of advanced but sophisticated LWW treatment technologies like advanced oxidation [51], electro-Fenton [52], membrane bioreactors [53], sensor-based systems [54], etc. in large scale agricultural settings. Moreover, further exploration into nutrient recovery potential from treated wastewater, comparison with conventional methods, sensitivity analyses, and strategies for knowledge transfer and adoption are crucial for advancing the application of RSM in LWW treatment [24,[55], [56], [57], [58]]. In addition, biological and physicochemical processes are often combined to treat highly polluted livestock waste streams [7,51,[59], [60], [61], [62]]. However, optimization of such combined treatment technologies using RSM has rarely been studied. Addressing these gaps would contribute significantly to the optimization and sustainable management of LWW using RSM. Considering the pertinency and significance of RSM, this review aims to consolidate and critically analyze the application of RSM in the context of LWW treatment. Specifically, this review not only synthesizes existing information regarding the current state of RSM applications in LWW treatment but also provides important insights into the RSM strategy, its application, associated challenges, potential areas of improvement, and future perspectives of RSM application in treating livestock waste streams.

## 2 Steps of response surface methodology

**Fig. 1:**
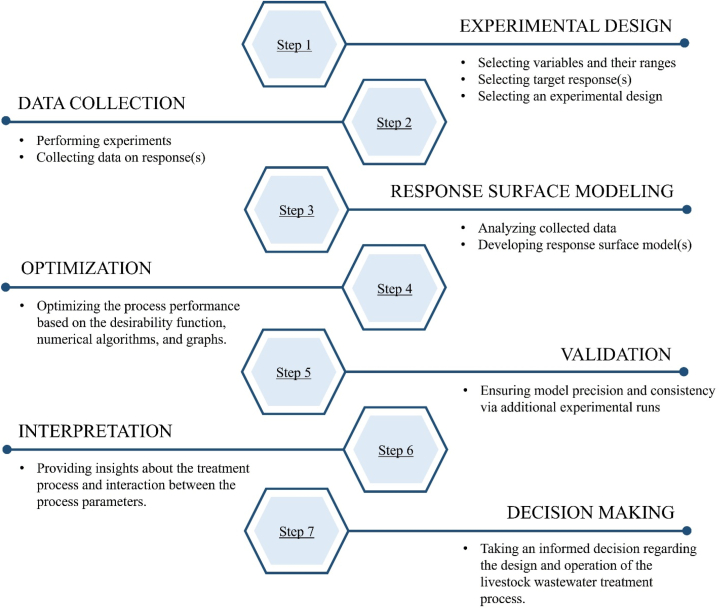
Schematic of the key steps involved in the RSM approach.

The RSM has a certain pattern involving several key steps. Fig. 1 represents a simplified outline of RSM. The steps indicated in Fig. 1 will help craft an experimental plan and be useful in reviewing and modifying certain perspectives of the research plan in LWW treatment. The details of the overall RSM approach are discussed in the following sub-sections.

### 2.1 Experimental design

**Fig. 2:**
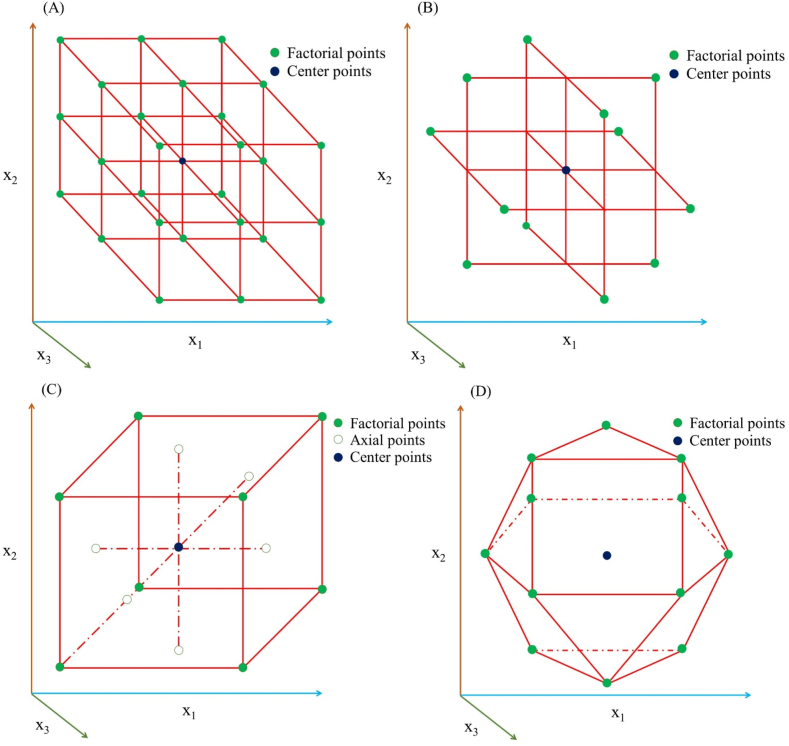
Different experimental designs for three variables at three levels (A) factorial design (B) Box-Behnken design (C) central composite design, and (D) Doehlert design.

The first step in RSM is to design and conduct experiments to collect data on the LWW treatment process performance. The experimental design should be carefully planned to ensure that the data obtained are statistically reliable and can be used to build accurate response surface models. Design of Experiments (DoE) techniques, such as factorial design, Box-Behnken design (BBD), central composite design (CCD), and Doehlert design (Fig. 2A–D), are commonly used in RSM to systematically vary the process parameters and collect data on the target response(s) [13]. Table 1 represents sample experimental matrices of aforementioned experimental designs having three variables at three levels. For example, while crafting an experimental design for ammonia removal from anaerobically digested liquid dairy manure using vacuum thermal stripping process factors such as temperature, vacuum pressure, and treatment time, are found to be most critical for efficient process operation [33,63]. The levels of these factors should therefore be selected on the basis of the expected range of operating conditions and available resources.

**Table 1:** Sample experimental matrices for (a) factorial, (b) Box-Behnken, (c) central composite, and (d) Doehlert designs (three variables at three levels; all values are shown in coded levels).

Run	(a)			(b)			(c)			(d)		
	x_1_	x_2_	x_3_	x_1_	x_2_	x_3_	x_1_	x_2_	x_3_	x_1_	x_2_	x_3_
1	−1	−1	−1	−1	−1	0	−1	−1	−1	0	0	0
2	0	−1	−1	1	−1	0	1	−1	−1	1	0	0
3	1	−1	−1	−1	1	0	−1	1	−1	0.5	0.866	0
4	−1	0	−1	1	1	0	1	1	−1	0.5	0.289	0.817
5	0	0	−1	−1	0	−1	−1	−1	1	−1	0	0
6	1	0	−1	1	0	−1	1	−1	1	−0.5	−0.866	0
7	−1	1	−1	−1	0	1	−1	1	1	−0.5	−0.289	−0.817
8	0	1	−1	1	0	1	1	1	1	0.5	−0.866	0
9	1	1	−1	0	−1	−1	−α	0	0	0.5	−0.289	−0.817
10	−1	−1	0	0	1	−1	α	0	0	−0.5	0.866	0
11	0	−1	0	0	−1	1	0	−α	0	0	0.577	−0.817
12	1	−1	0	0	1	1	0	α	0	−0.5	0.289	0.817
13	−1	0	0	0	0	0	0	0	−α	0	−0.577	0.817
14	0	0	0				0	0	α			
15	1	0	0				0	0	0			
16	−1	1	0									
17	0	1	0									
18	1	1	0									
19	−1	−1	1									
20	0	−1	1									
21	1	−1	1									
22	−1	0	1									
23	0	0	1									
24	1	0	1									
25	−1	1	1									
26	0	1	1									
27	1	1	1									
28	0	0	0									

### 2.2 Data collection

Once the experimental design has been finalized, the experiments are performed and data on the response variables are recorded. It is critical to ensure that the collected data are accurate and reliable as well as free of any sources of variability or measurement errors [64]. Data on the response variable(s) collected under various experimental conditions are used to identify the optimal set of parameters that lead to the desired outcome, such as maximum pollutant removal or minimum resource consumption [65,66]. To effectively describe the behavior of the particular LWW treatment process and develop the RSM model, the experimentally obtained data should cover a wide range of operating conditions.

### 2.3 Response surface modeling

**Fig. 3:**
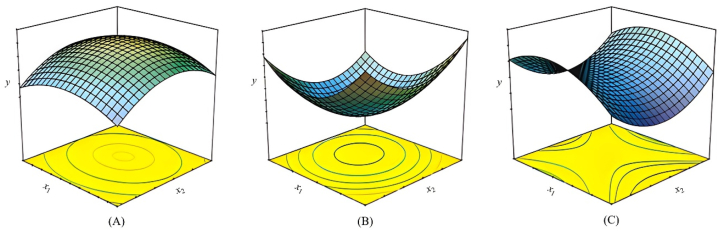
Different types of three-dimensional surface and contour plots generated from a quadratic model, (A) maximum, (B) minimum, and (C) saddle.

The next step is to analyze the collected data and construct response surface models, which are mathematical models that explain the relationship between the process parameters and the target response variable(s). The most basic model used in RSM is based on a linear function [67], and to apply it successfully the obtained experimental results must adapt well to Eq. (1). Consequently, the responses ought to be devoid of any curvature. To assess curvature, a second-order model becomes necessary. Two-level factorial designs are effective for estimating first-order effects but do not account for significant additional effects, such as second-order effects. A central point in two-level factorial designs can be used to account for curvature and results in a second-order polynomial interactive model that depicts the interaction between the independent variables (Eq. (2)) [68]. Furthermore, quadratic terms can be incorporated into the second-order polynomial function (Eq. (3)) to establish a critical point (maximum, minimum, or saddle, Fig. 3A–C) [11]. To calculate the parameters in Eq. (3), the experimental design must ensure that all the independent variables are examined at a minimum of three-factor levels. As a result, two symmetrical response surface designs are available for modeling purposes. Examples of second-order symmetrical designs include the three-level factorial design, BBD, CCD, and Doehlert design, among others. These designs differ in terms of their selection of experimental points (factorial, axial, and central), number of levels for variables, and number of runs and blocks.

(1) $$y={\;\beta }_{0}+\sum_{i}^{k}\beta_{i}x_{i}+\varepsilon$$

(2) $$y=\beta_{0}+\sum_{i=1}^{k}\beta_{i}x_{i}+\sum_{1\leq i\leq j}^{k}\beta_{\text{ij}}x_{i}x_{j}+\varepsilon$$

(3) $$y=\beta_{0}+\sum_{i=1}^{k}\beta_{i}x_{i}+\sum_{i=1}^{k}\beta_{\text{ii}}x_{i}^{2}+\sum_{1\leq i\leq j}^{k}\beta_{\text{ij}}x_{i}x_{j}+\varepsilon$$

where y is the predicted response, β_0_ represents the constant term, k indicates the number of variables, β_i_ denotes the coefficients of the linear parameters, x_i_ represents the variables, β_ii_ depicts the coefficients of interaction between parameters x_i_ and x_j_, β_ij_ symbolizes the coefficients of quadratic terms, and ɛ is the residual associated to the experiments.

These models can help understand the complex interactions between the process parameters and the response variable(s) and provide insights into the optimal operating conditions for the LWW treatment process.

### 2.4 Model evaluation

The model evaluation is a crucial step in RSM as it confirms the reliability and prediction ability of the developed response surface models under different conditions. Among different statistical methods, the analysis of variance (ANOVA) is the most widely used to assess the predictive performance of the response surface models and determine their validity as it compares variation during the experimental runs with the variability caused by the random errors that are naturally found in the obtained experimental values of the target response(s) based on the deviation analysis [43]. Such a comparison elucidates the significance of the regression employed in predicting responses considering the factors that contribute to experimental variability. The degree of deviation (d_i_) exhibited by each observation ($y_{i}$) or its duplicates ($y_{\text{ij}}$) from the mean value ($\bar{y}$) and can be expressed as the square of this deviation (Eq. (4))):

(4) $$d_{i}^{2}={\left(y_{\text{ij}}\;-\;\bar{y}\right)}^{2}$$

The total sum of squares (${\text{SS}}_{\text{tot}}$) represents the sum of the squared deviations of all observations from the mean. It has two components: the sum of squares due to the fitted mathematical model, or regression (${\text{SS}}_{\text{reg}}$), and the sum of squares due to the residuals generated by the model (${\text{SS}}_{\text{res}}$), as shown in Eq. (5):

(5) $${\text{SS}}_{\text{tot}}={\text{SS}}_{\text{reg}}+{\;\text{SS}}_{\text{res}}$$

Furthermore, replicating the central point makes it possible to calculate the pure error associated with repetitions. Consequently, the ${\text{SS}}_{\text{res}}$ can be further divided into two components: the sum of squares due to pure error (${\text{SS}}_{\text{pe}}$) and the sum of squares due to lack of fit (${\text{SS}}_{\text{lof}}$), as presented in Eq. (6):

(6) $${\text{SS}}_{\text{res}}={\text{SS}}_{\text{pe}}+{\;\text{SS}}_{\text{lof}}$$

To obtain the mean square (MS) for each source of variation (total, regression, residual, lack of fit (lof), and pure error (pe)), the sum of square (SS) is divided by their respective degrees of freedom (dof). The equations related to the calculation of sum of squares (SSs) and mean squares (MSs) for each source of variation are listed in Table 2 [69,70].

**Table 2:** Analysis of variance (ANOVA) for a fitted mathematical model to an experimental data set using multiple regression.

Parameter	Sum of square	Degree of freedom	Mean of square
Regression	${\text{SS}}_{\text{reg}}=\sum_{i}^{m}\sum_{j}^{n_{1}}{\left({\hat{y}}_{i}‐\bar{y}\right)}^{2}$	p – 1	${\text{MS}}_{\text{reg}}=\frac{{\text{SS}}_{\text{reg}}}{p\;–\;1\;}$
Residuals	${\text{SS}}_{\text{res}}=\sum_{i}^{m}\sum_{j}^{n_{1}}{\left(y_{\text{ij}}‐{\hat{y}}_{i}\right)}^{2}$	n – p	${\text{MS}}_{\text{res}}=\frac{{\text{SS}}_{\text{res}}}{n\;–\;p\;}$
Lack of fit	${\text{SS}}_{\text{lof}}=\sum_{i}^{m}\sum_{j}^{n_{1}}{\left({\hat{y}}_{i}‐{\bar{y}}_{i}\right)}^{2}$	m – p	${\text{MS}}_{\text{lof}}=\frac{{\text{SS}}_{\text{lof}}}{m\;–\;p\;}$
Pure error	${\text{SS}}_{\text{pe}}=\sum_{i}^{m}\sum_{j}^{n_{1}}{\left(y_{\text{ij}}‐{\bar{y}}_{i}\right)}^{2}$	n – m	${\text{MS}}_{\text{pe}}=\frac{{\text{SS}}_{\text{pe}}}{n\;–\;m\;}$
Total	${\text{SS}}_{\text{tot}\;}=\sum_{i}^{m}\sum_{j}^{n_{1}}{\left(y_{\text{ij}}‐\bar{y}\right)}^{2}$	n – 1	

n_i_, number of observations; m, number of levels (total) in the design; p, number of model parameters; ${\hat{y}}_{i}$, estimated value by the model for the level i; $\bar{y}$, overall mean; y_ij_, replicates performed in respective individual levels; ${\bar{y}}_{i}$, means of replicates conducted in the same set of experimental conditions.

The significance of regression can be assessed comparing the mean square of regression (MS_reg_) to the mean square of residuals (MS_res_) and using the Fisher distribution test (F test), considering the corresponding dof associated with regression ($\upsilon_{\text{reg}}$) and residual ($\upsilon_{\text{res}}$) variances (Eq. (7)):

(7) $$\frac{{\text{MS}}_{\text{reg}}}{{\text{MS}}_{\text{res}}\;}\approx F_{\upsilon_{\text{reg}},\upsilon_{\text{res}}}$$

In this context, a statistically significant value for this ratio should exceed the tabulated F-value. This indicates that the mathematical model fits well with the experimental observations.

Another approach to evaluate the model is the dof test. If the mathematical model fits well with the experimental data, mean squares lack of fit (MS_lof_) should primarily reflect random errors inherent in the system. Additionally, MS_pe_ serves as an estimate of these random errors, assuming that these two values are not statistically different. This forms the foundation of the lof test. The F distribution can be used to determine whether there is a statistical difference between these two means, similar to the significance of regression (Eq. (8)):

(8) $$\frac{{\text{MS}}_{\text{lof}}}{{\text{MS}}_{\text{pe}}\;}\approx F_{\upsilon_{\text{lof}},\upsilon_{\text{pe}}}$$

where $\upsilon_{\text{lof}}$ and $\upsilon_{\text{pe}}$ represent the *dof* associated with *lof* and *pe* variances, respectively. If this ratio exceeds the tabulated F-value, it indicates evidence of lof and the need for model improvement. Conversely, if the value is lower than the tabulated value, the model's fitness can be considered satisfactory. To conduct a lof test, the experimental design must include actual replications, at least at its central point.

In summary, a well-fitted model accurately describes the experimental data when it exhibits a significant regression and a non-significant lof. To be more precise, most of the observed variations are ascribed by the regression equation, while the remaining variations are attributed to residuals. The variations related to residuals are primarily due to random measurement errors (pe), rather than lof, which directly relates to the quality of the model [71,72].

The visual examination of residual graphs can also provide valuable insights into the suitability of the model. If the mathematical model fits well, the residual graph demonstrates behavior indicative of a normal distribution. Conversely, if the model generates larger residuals, it is not suitable for making precise inferences about the data's behavior within the studied experimental domain. Furthermore, if the model requires additional terms, the residual graph will exhibit behavior suggesting the type of term that should be included in the model [69].

### 2.5 Model interpretation

Following evaluation, the developed response surface models need to be interpreted to gain insights into the behavior of the respective LWW treatment process and the relationships between the process parameters and the response variable. Model interpretation involves analyzing the coefficients or parameters of the models as well as the plots or surfaces generated from the models, to understand the direction and magnitude of the effects of the process parameters on the response variable(s). Model interpretation provides insights into the underlying mechanisms of the treatment process, helps identify the most important influential factors affecting the process performance, and guides the selection of optimal operating conditions.

### 2.6 Optimization

Once the response surface models are constructed, the next step is to optimize the LWW treatment process to achieve the desired treatment performance. The optimization process involves exploring different combinations of process parameters within the feasible range defined by experimental design and available resources. The optimal operating conditions should be selected on the basis of the response surface model results and validated using additional experiments or independent data.

Different optimization techniques, such as desirability function approach, numerical optimization algorithms, or graphical optimization, can be employed to identify the optimal operating conditions that maximize the response variable or achieve other specific goals, such as meeting effluent quality standards, minimizing operating costs, or maximizing resource utilization. The simplicity of a desirability function approach has led to its extensive use in process optimization among the aforementioned optimization techniques [73]. The desirability function approach converts a predicted response into a dimensionless value known as desirability [[74], [75], [76]]. This approach allows optimization objectives to be aimed at maximizing, minimizing, or achieving a target value for the response [77]. Various desirability functions can be utilized depending on the specific work objective. Derringer and Suich (1980) introduced a specific function to convert target responses ($y_{i}$) to desirability ($d_{i}\left(y_{i}\right)$) using the following two types of transformations:

The first transformation is employed to minimize or maximize y_i_ and the second transformation is applied to achieve the desired target value ($y_{i}$) for $y_{i}$, with $l_{i}$ representing the lower bound and u_i_ indicating the upper bound of the $y_{i}$ (Eq. (9)).

The powers s and t are associated with the weighting factor, influencing the shape of the desirability function, d_i_(y_i_). The values of s and t act as parameters that determine the specific curvature of the function. When s = t = 1, the desirability function increases linearly toward t_i_. If s < 1 and t < 1, the function exhibits a concave shape. Conversely, if s > 1 and t > 1, the function takes on a convex form [76,77].

(9) $$d_{i}\left(y_{i}\right)=\left\{ \begin{array}{c} 0,\text{yi}<\text{li}\\ {\left(\frac{y_{i}\;–\;l_{i}}{t_{i}\;–\;l_{i}}\right)}^{s},l_{i}\leq y_{i}\leq t_{i}\;\\ {\left(\frac{y_{i}\;–\;u_{i}}{t_{i}\;–\;u_{i}}\right)}^{t},t_{i}\leq y_{i}\leq u_{i}\\ 0,y_{i}>u_{i} \end{array}\right.$$

The complete desirability function (D) is the geometric mean of the individual desirability functions for each response, $d_{i}\left(y_{i}\right)$ (Eq. (10)), where n represents the number of responses. The optimal solutions are identified by maximizing the value of D [76].

(10) D=(∏i=1ndi(yi))1n

### 2.7 Validation of optimized conditions

The validation of suggested optimized conditions obtained through the RSM approach is a decisive step to ensure the reliability and effectiveness of the model. By conducting experiments based on the suggested conditions, researchers can assess whether the predicted outcomes align with actual observations. During the validation process, the experimental setup is designed according to the recommended optimal settings derived from the RSM model. Depending on the LWW treatment technology, the process parameters are carefully controlled and adjusted. The obtained experimental results are then compared to the predicted values generated by the RSM model. If the average of the experimental values closely matches the predicted values or falls between 95 % low and high confidence interval values, it provides evidence that the RSM model is accurate and reliable in optimizing the desired response [34]. On the other hand, significant discrepancies between the predicted and observed values may indicate the need for further adjustments or refinements in the model. Overall, validation of RSM suggested optimized conditions ensures the practical applicability and effectiveness of the model, providing researchers with confidence in utilizing the optimized conditions for future experiments or industrial applications.

To sum up, the RSM methodology in LWW treatment involves a systematic approach consisting of experimental design, data collection, response surface modeling, model evaluation, model interpretation, process optimization, and validation. This approach helps optimize the performance of the LWW treatment process, realize the complex interactions between process parameters and the response variable(s), and make informed decisions regarding the design and operation of the system.

## 3 Application of RSM in various livestock wastewater treatment processes

The application of RSM in LWW processes has gained significant attention in recent years due to the increasing concerns about environmental pollution and the need for sustainable wastewater management practices in the livestock industry. RSM, as a statistical and mathematical tool, provides a systematic approach to optimize the performance of wastewater treatment processes by identifying the key factors and their interactions that affect treatment efficiency. This section provides an overview of RSM application in various LWW treatment processes.

### 3.1 Biological treatment processes

Anaerobic digestion (AD) is the most commonly employed biological LWW treatment process, as it can effectively remove organic matter and generate biogas as a renewable energy source (Table 3). Despite this, biogas production from livestock waste steams through anaerobic processes is not regarded as a cost-effective option due to its low methane (CH_4_) production potential [78,79]. Therefore, researchers suggest that the co-digestion (CD) of livestock waste streams with other feedstock would be economically viable and environmentally sustainable as the process can improve CH_4_ yields [[80], [81], [82], [83], [84]]. A recent study reported that maximum CH_4_ production can be increased up to 35.2–58.0 % when co-digested with other feedstocks [85]. Such implications highlight the importance of optimizing AD and CD processes to treat LWW. Compared to the conventional optimization approach (OFAT), RSM-optimized systems showed better performance in terms of CH_4_ yield during AD and CD of LWW [86,87]. Hence, several studies applied RSM to optimize key operating parameters such as temperature, hydraulic retention time (HRT), pH, substrate concentration, mixing ratio, total solids content, and treatment time in anaerobic digestion and co-digestion of LWW to achieve maximum biogas production along with organic matter removal efficiency (Table 3). Moreover, biological processes have also used RSM to optimize nutrient removal efficiencies from LWW. Amini et al. [40] developed a batch system simultaneous nutrient removal process to treat dairy wastewater and optimized the process parameters such as mixed liquor suspended solids, COD/N and COD/P ratios, aeration rate, and cycling time using CDD-RSM approach to achieve maximum nutrient removal efficiency. Furthermore, Gadhe et al. [88] employed a BBD-RSM design to increase the hydrogen production potential from swine wastewater by optimizing substrate concentration, pH, COD/N, and COD/P ratios. Along with improving biogas productivity and pollutant removal efficiency, optimization of biological LWW treatment processes can help evaluate the process performance, save time, and reduce operational costs [87,89]. So, RSM can serve as an effective tool to investigate the interactions between different factors and their impacts on biogas production, nutrient removal, and process stability in biological LWW treatment.

**Table 3:** Some recent applications of RSM in biological LWW treatment processes.

Treatment process	RSM approach	Experimental runs	Optimized process parameters	Target responses	References
Swine wastewater/manure					
Co-digestion with vegetable processing waste	CCD	9	1. Substrate concentration (Volatile solids as g VS L–1) 2. Proportion of VPW mass (%)	1. Volatile solids removal (%) 2. Methane production (mL CH_4_ g^−1^ VS added)	[90]
Co-digestion with microalgal biomass	CCD	9	1. COD/VS ratio 2. % COD algae	1. Methane yield (mL CH_4_ g COD_in_^−1^) 2. TS removal (%)	[91]
Co-digestion with food waste leachate	CCD	20	1. Mixing ratio 2. Alkalinity (mg CaCO3 L−1) 3. Salinity (g NaCl L−1)	1. Volatile solids reduction (%) 2. Methane production (mL CH_4_ g^−1^ VS added)	[92]
Co-digestion with food waste	CCD	11	1. Mixing ratio 2. Particle size (cm)	1. Methane production (mL CH_4_ g^−1^ VS added) 2. C/N ratio	[93]
Acidogenically digested swine manure-based algal system	CCD	13	1. Dilution rates (times) 2. Hydraulic retention time (days)	1. Biomass productivity (mg L^−1^ d^−1^) 2. COD (mg L^−1^ d^−1^) 3. PO_4_–P (mg L^−1^ d^−1^) 4. TN (mg L^−1^ d^−1^) 5. NH_3_–N (mg L^−1^ d^−1^)	[94]
Hydrogen production from co-fermenting molasses with liquid swine manure	CCD	20	1. pH 2. Hydraulic retention time (h) 3. TS content (%)	1. Biogas production rate (L d^−1^) 2. Hydrogen production rate (L^−1^ d^−1^ L^−1^) 3. Hydrogen content (%) 4. Hydrogen yield (mol-H_2_ mol^−1^-sugar)	[95]
Two-step fed sequencing batch reactor system	CCD	20	1. First/second feed ratio (mL/mL) 2. First an/oxic ratio (min/min) 3. Second an/oxic ratio (min/min)	1. TN (%) 2. NH_4_–N (%) 3. TP (%) 4. DP (%) 5. COD (%) 6. BOD (%)	[42]
Microalgae production	CCD	20	1. Temperature (°C) 2. Light intensity (μE m^−2^ s^−1^) 3. pH	1. Biomass concentration (g L^−1^)	[39]
Co-digestion with peanut hulls	CCD	23	1. TS content (%) 2. C/N ratio 3. Inoculum volume (%)	1. Gas production (mL g^−1^ VS) 2. Methane production (mL CH_4_ g^−1^ VS)	[96]
Co-digestion with corn stalks	CCD	20	1. TS content (%) 2. Amount of added biochar (%) 3. C/N ratio	1. Biogas production rate (mL g^−1^ VS)	[97]
Anaerobic digestion	CCD	11	1. pH 2. Time (d)	1. Methane production rate (mL L^−1^d^−1^) 2. Methane yield (mL g^−1^ VS_add_)	[41]
Co-digestion with rice straw under the presence of sycamore sawdust biochar	BBD	17	1. TS (%) 2. Swine manure (%TS) 3. Biochar (% TS)	1. Methane yield (mL g^−1^ VS_add_)	[98]
Immobilized bacteria pellet mediated NH_3_–N removal	CCD	20	1. Sodium alginate dosage 2. Chitosan dosage 3. Embedding time	1. NH_3_–N removal (%)	[99]
Co-digestion with *Prosopis juliflora* seeds, water hyacinth, and dry leaves	CCD	10	1. Temperature (°C) 2. pH	1. Methane yield (%)	[100]
Anaerobic digestion	CCD	29	1. Hydraulic retention time (d) 2. Temperature (°C) 3. Moisture content (%) 4. pH 5. C/N ratio	1. Methane yield (%)	[101]
Dairy wastewater/manure					
Co-digestion with vegetable processing waste	CCD	9	1. Substrate concentration (Volatile solids as g VS L^−1^) 2. Proportion of VPW mass (%)	1. Volatile solids removal (%) 2. Methane production (mL CH_4_ g^−1^ VS added)	[90]
Hydrogen production	BBD	27	1. Substrate concentration (g COD L^−1^) 2. pH 3. COD/N ratio 1. COD/P ratio	1. Hydrogen yield (H_2_ mmol g^−1^ COD) 2. Specific hydrogen production rate 3. (H_2_ mmol g^−1^ VSS d^−1^)	[88]
Batch system for simultaneous COD and N removal	CCD	30	1. Mixed liquor suspended solids (mg L^−1^) 2. COD/N ratio 3. COD/P ratio 4. Aeration rate (min h^−1^) 5. Cycling time (h)	1. COD removal (%) 2. TKN removal (%) 3. N–NO_3_^–^ removal (%) 4. N–NO_3_^–^ effluent (mg L^−1^) 5. TN effluent (mg L^−1^)	[40]
Sequencing batch reactor system under simultaneous aerobic/anaerobic conditions	CCD	30	1. Mixed liquor suspended solids (mg L^−1^) 2. COD/N ratio 3. Aeration rate (min h^−1^) 4. Cycling time (h)	1. PO_4_^3−^ removal (%) 2. Sludge volume index (mL g^−1^) 3. Mixed liquor volatile suspended solids (mg L^−1^) 4. Mixed liquor suspended solids (mg L^−1^)	[102]
Anaerobic digestion	CCD	29	1. Organic loading rate (kg VS m^−3^day^−1^) 2. Temperature (°C) 3. Mixing rate (rpm)	1. Biogas production rate (m^3^ m^−3^ day^−1^) 2. Biogas yield (m^3^ kg^−1^ VS added) 3. Methane production rate (m^3^ m^−3^ day^−1^) 4. Methane yield (m^3^ kg^−1^ VS added)	[103]
Co-digestion with rice straw	CCD	30	1. Temperature (°C) 2. pH 3. Substrate amount (kg) 4. Agitation time (s)	1. Biogas yield (L)	[104]
Co-digestion with waste food materials	BBD	13	1. Temperature (°C) 2. pH 3. Solid to water ratio	1. Volume of methane produced (mL)	[105]
Anaerobic digestion	CCD	10	1. Sonication time (min) 2. Slurry ratio 3. Digestion time (day)	1. Volume of biogas produced (mL)	[106]
Co-digestion of corn-chaff	CCD	13	1. Mixing ratio 2. Hydraulic retention time (d)	1. Cumulative biogas yield (L)	[107]
Co-digestion with pineapple waste	CCD	20	1. Temperature (°C) 2. Reaction time (min) 3. Water to solid ration	1. Biogas yield (mL g^−1^ VS)	[108]
Microalgal treatment	CCD	16	1. Dairy wastewater concentration (%) 2. Temperature (°C) 3. Light intensity (lx)	1. BOD removal (%) 2. COD removal (%) 3. Biomass concentration (g L^−1^)	[109]
Microbial fuel cell	CCD	11	1. Aeration (mL min^−1^) 2. Yeast extract (g L^−1^)	1. COD removal (%) 2. Power density (mW m^−2^)	[110]
Intermittently-aerated-extended-idle sequencing batch reactor	CCD		1. Cycle-time (h) 2. Intermittent-aeration rate (min/h) 3. Feed-phases (min) 4. Idle-phase (min)	1. O–P removal (%) 2. TP removal (%) 3. NH_3_–N removal (%) 4. TN removal (%) 5. COD removal (%)	[111]
Poultry manure					
Anaerobic digestion	CCD	13	1. Agitation (rpm) 2. Reaction time (d)	1. Biogas yield (L g^−1^ COD)	[112]
Anaerobic co-digestion with citrus pulp and lawn grass	CCD	15	1. Citrus pulp (% TS) 2. Lawn grass (% TS) 3. Chicken manure (% TS)	1. Biogas production (mL)	[113]
Anaerobic digestion	CCD	30	1. TS content (%) 2. Inoculum ration (%) 3. Amount of pumice (g L^−1^) 4. C/N ratio	1. Cumulative biogas production (mL) 2. CH_4_ content (%) 3. COD removal (%)	[114]
Fermentation	CCD	27	1. Substrate–inoculum ratio 2. Incubation time (d) 3. TS content (%) 4. NaCl concentration (%)	1. Methane yield (mL g^−1^ VS)	[38]
Anaerobic digestion	CCD	20	1. Manure loading (g VS L^−1^) 2. Biochar dosage (%) 3. Cellulose loading (g VS L^−1^)	1. Cumulative methane yield (mL g^−1^ VS)	[115]
Anaerobic digestion	CCD	13	1. Sonication time (min) 2. Slurry ratio	1. Volume of biogas produced (mL)	[116]
Microbial fuel cell	CCD	20	1. Electrode distance (cm) 2. Moisture content (%) 3. Temperature (°C)	1. Biomass (mL L^−1^) 2. COD removal (%) 3. Power density (mW m^−2^)	[117]
Anaerobic digestion	CCD	20	1. TS content (%) 2. Ni ratio loaded on TP (w/w %) 3. Ni/TP amount (mg L^−1^)	1. Cumulative biogas production (mL) 2. CH_4_ content (%) 3. COD removal (%)	[118]
Mixed manure					
Anaerobic co-digestion of dairy manure, chicken manure, and wheat straw	CCD	9	1. Dairy manure/chicken manure ratio 2. C/N ratio	1. Methane potential (mL kg^−1^ VS)	[119]
Anaerobic co-digestion of dairy, chicken, swine manure, and wheat straw	CCD	13	1. Manure ratio 2. C/N ratio	1. Methane potential (mL g^−1^ VS)	[120]
Anaerobic co-digestion of cow dung, chicken manure, and sugar beet root waste	CCD	12	1. Cow dung mass fraction 2. C/N ratio	1. Methane yield (mL g^−1^ VS)	[121]

### 3.2 Physical treatment processes

Physical LWW treatment processes are essential in mitigating the environmental impact of livestock operations. These processes involve a range of techniques aimed at removing suspended solids, organic matter, nutrients, and other contaminants from wastewater. Different physical treatment technologies including physical sedimentation, filtration, screening, screw press, settling basin, and centrifugation are generally used in treating highly polluted livestock waste streams [[122], [123], [124], [125]]. However, RSM in terms of physical LWW treatment technologies is mainly used to optimize more advanced [30] processes like air stripping, thermal stripping along with other pretreatment practices (Table 4). For instance, ammonia emission from swine wastewater is a known health hazard for farmers and can also disrupt animal growth [126,127]. Air stripping is one of the widely adopted ammonia inhibition technologies due to its higher removal efficiencies [128]. Cao et al. [129] observed more than 85 % ammonia removal efficiencies from swine wastewater using air stripping within the pH range of 7–9 at 45 °C. Whereas Liu et al. [130] reported that around 97 % of ammonia can be removed from swine wastewater upon optimization of the air stripping process using RSM. Recently, the vacuum thermal stripping-acid absorption process has gained popularity among researchers owing to its high removal and recovery potential. When the system was first developed by Ukwuani and Tao [131], more than 93 % of ammonia was removed from anaerobically digested liquid dairy manure after 180 min of treatment time. Later, Reza and Chen [33] improved and employed RSM to optimize the treatment process and obtained identical removal efficiency within 88 min of process operation. Similar augmentation in process performance were also reported by researchers for other physical treatment technologies upon optimization using RSM [31,32,132,133]. As such, it can be said that RSM-optimized processes not only perform efficiently in removing pollutants from LWW but also help conserve resources.

**Table 4:** Some recent applications of RSM in physical LWW treatment processes.

Treatment process	RSM approach	Experimental runs	Optimized process parameters	Target responses	References
Swine wastewater/manure					
Air-recirculated stripping	BBD	15	1. Gas flow rate (L min^−1^) 2. Lime dose (g) 3. Gas-to-liquid ratio	1. NH_4_^+^-N removal (%)	[30]
Aqueous ammonia soaking pretreatment	CCD	19	1. NH_3_ concentration (% w/w) 2. Treatment duration (hrs) 3. S:L ratio (kg fibers L^−1^ reagent)	1. COD solubilization (%) 2. CH_4_ yield 17d (mL CH_4_ g^−1^ TS) 3. Increase in CH_4_ yield 17d (%)	[31]
Alkaline microwaving pretreatment	CCD	10	1. pH 2. Energy (J g^−1^)	1. Disintegration degree (%)	[132]
Microwave assisted digestion	CCD	30	1. Microwave temperature (K) 2. Microwave time (min) 3. H_2_O_2_ addition amount (mL 30 mL^−1^) 4. HCl addition percentage (%)	1. IP (mg L^−1^) 2. TP (mg L^−1^)	[32]
Dairy wastewater/manure					
Microwave irradiated thermal stripping	CCD	20	1. pH 2. Irradiation time (min) 3. Power output (W)	1. NH_3_ removal (%)	[133]
Vacuum thermal stripping	CCD	20	1. Temperature (°C) 2. Vacuum pressure (kPa) 3. Treatment time (min)	1. NH_3_ removal (%)	[33]

### 3.3 Chemical treatment processes

Chemical treatment methods offer effective means of treating LWW to remove pollutants and improve its quality before discharge into the environment or reuse. Chemical treatment methods are usually applied in combination with physical and biological treatments to achieve maximum efficiency [134,135] or as a standalone process [13,[136], [137], [138], [139], [140], [141]]. The most widely used chemical LWW treatment methods include precipitation, coagulation and flocculation, pyrolysis, electrocoagulation, adsorption, and Fenton processes, and can be optimized using RSM for better process performance and resource conservation (Table 5). For example, chemical precipitation methods are employed to remove nutrients and trace metals from livestock wastewater. Among the nutrients present in LWW, P removal and recovery can be achieved via struvite [e.g., magnesium ammonium phosphate hexahydrate (MgNH_4_PO_4_·6H_2_O)] precipitation through the addition of Mg sources, which is sparingly dissolved in neutral and alkaline pH, but readily dissolves in acidic conditions [142]. The RSM-optimized struvite crystallization process can improve struvite recovery efficiency and production rate together with particle size [13,15]. Electrochemical treatment, another popular LWW management approach that has attracted considerable attention in recent years. Among different electrochemical treatment technologies, electrocoagulation is most widely used due to its simplicity and ability to treat a wide array of pollutants including emerging contaminants present in LWW [138,[143], [144], [145], [146], [147]]. Regardless of electrode materials, parameters such as current density, pH, treatment time, electrolyte concentration, and interelectrode gaps are important for the successful and efficacious operation of electrocoagulation processes [[148], [149], [150], [151]]. Studies posited that optimization of the above-mentioned process parameters using RSM can significantly improve pollutant removal efficiencies and alleviate LWW treatment costs [138,147,152]. These findings are also applicable to other chemical LWW treatment technologies like adsorption, Fenton, and pyrolysis processes. As such, improvement in pollutant removal performance and minimization of resource utilization together with cost reduction can be achieved through systematic application of RSM in chemical LWW treatment processes.

**Table 5:** Some recent applications of RSM in chemical LWW treatment processes.

Treatment process	RSM approach	Experimental runs	Optimized process parameters	Target responses	References
Swine wastewater/manure/slaughterhouse wastewater					
Phosphorus recovery using chemical precipitation	CCD	30	1. pH 2. NH_4_^+^–N concentration (mg L^−1^) 3. PO_4_^3^–P concentration (mg L^−1^) 4. Mg concentration (mg L^−1^) 5. Ca concentration (mg L^−1^)	1. Recovery efficiency (%) 2. Recovered P (mg L^−1^)	[15]
Struvite recovery using fluidized bed reactor	CCD	32	1. pH 2. P concentration (mg L^−1^) 3. Mg concentration (mg L^−1^) 4. Upflow velocity (mm s^−1^) 5. Recycle ratio (%)	1. SOP removal (%) 2. TP removal (%)	[153]
Electrocoagulation	CCD	20	1. Electrolysis time (min) 2. Current density (A m^−2^) 3. pH	1. COD removal (%)	[154]
Struvite precipitation	CCD	29	1. pH 2. Mg:P molar ratio 3. Ca:Mg molar ratio 4. CO_3_^2−^:P molar ratio	1. P recovery efficiency (%)	[155]
Struvite recovery using fluidized bed reactor	CCD	18	1. pH 2. Circulation rate (L L_reactor_^−1^ h^−1^) 3. Hydraulic retention time (h)	1. OP removal (%) 2. NH_4_–N removal (%) 3. (NH_3_)_stripping_ (%) 4. (NH_4_^+^)_struvite_ (%) 5. Particle size (μm) 6. Struvite production rate (kg struvite m^−3^_reactor_ d^−1^)	[13]
Struvite crystallization	CCD	9	1. pH 2. Mg:P molar ratio	1. Struvite crystallization efficiency (%)	[156]
Fenton process	CCD	20	1. H_2_O_2_ concentration (mg L^−1^) 2. FeSO_4_ concentration (mg L^−1^) 3. Time (min)	1. TOC removal (%) 2. Color removal (%)	[34]
Adsorption	CCD	30	1. pH 2. Cu & Zn concentration (μM) 3. Cu/Zn ratio 4. HCO_3_^−^ concentration (mM)	1. Cu adsorption capacity (mg L^−1^) 2. Zn adsorption capacity (mg L^−1^) 3. P recovery rate (%)	[157]
Dairy wastewater/manure					
Electrocoagulation	CCD	30	1. Current density (A m^−2^) 2. Salt dosage (g L^−1^) 3. Time (min) 4. pH	1. COD removal (%)	[158]
Electrocoagulation	CCD		1. pH 2. Current density (A m^−2^) 3. Time (min)	1. COD removal (%) 2. Color removal (%) 3. O–P removal (%) 4. TSS removal (%) 5. Turbidity removal (%)	[37]
Electrocoagulation	CCD	30	1. pH 2. Electric current (A) 3. Time (min)	1. Turbidity removal (%) 2. Color removal (%) 3. COD removal (%)	[159]
Electrocoagulation	CCD	9	1. pH 2. Interelectrode distance (cm)	1. COD removal (%)	[160]
Electrocoagulation	BBD	17	1. Current density (mA cm^−2^) 2. pH 3. Conductivity (μS cm^−2^)	1. COD removal (%) 2. Anode consumption (mg mg^−1^ COD) 3. Energy consumption (J mg^−1^ COD)	[161]
Electrocoagulation	CCD	14	1. Residence time (min) 2. Elapsed time (min)	1. COD removal (%) 2. Energy consumed (kWh kg^−1^ COD removed)	[162]
Flocculation	CCD	13	1. Chitosan (mg L^−1^) 2. Kaolin (mg L^−1^)	1. Turbidity removal (%) 2. COD removal (%) 3. TKN removal (%) 4. TP removal (%)	[35]
Fenton process	CCD	24	1. pH 2. H_2_O_2_ concentration (mol L^−1^) 3. FeSO_4_ concentration (mol L^−1^) 4. Reaction time (min)	1. COD removal (%) 2. Turbidity removal (%)	[163]
Straw-derived biochar coupled with Mg/La oxides	BBD	17	1. Concentration (mg L^−1^) 2. Flow velocity (mL min^−1^) 3. Column height (cm)	1. Equilibrium adsorption capacity (mg g^−1^)	[164]
Poultry manure/litter/slaughterhouse wastewater					
Pyrolysis	CCD	20	1. Temperature (°C) 2. Heating rate (°C min^−1^) 3. Reaction time (min)	1. Biochar surface area (m^2^ g^−1^)	[36]
Pyrolysis	CCD	30	1. Poultry litter biochar mass (g) 2. pH 3. Stirring time (h) 4. Cd^2+^ concentration (mg L^−1^)	1. Cd^2+^ reduction (%)	[165]
Electrocoagulation and peroxidation	CCD	30	1. pH 2. Current density (mA cm^−2^) 3. Contact time (min) 4. H_2_O_2_ dosage (g L^−1^)	1. COD removal (%) 2. TSS removal (%) 3. Color removal (%)	[166]

In conclusion, RSM has been widely applied in various LWW treatment processes to optimize the performance of these processes and achieve maximum treatment efficiency. By identifying the key factors and their interactions, RSM provides a systematic approach to optimize the operational parameters of LWW treatment processes, resulting in improved treatment efficiency, reduced resource consumption, and more sustainable wastewater management practices in livestock industry.

## 4 Challenges associated with RSM application in livestock wastewater treatment

### 4.1 Simplified modeling assumptions

Response surface methodology relies on simplified mathematical models to represent the relationship between the input variables and the response variable(s). These models may not fully capture the complex and dynamic nature of LWW processes, which involve various biological, chemical, and physical reactions [23,25]. The assumptions made in the modeling process, such as linearity and normality of the response surface, may not always hold true in practice, leading to potential limitations in the accuracy and reliability of the optimization results [167]. Moreover, RSM typically assumes a static or steady-state condition [168] and may not adequately account for process dynamics. This can result in suboptimal solutions, as the optimal operating conditions may vary over time. Therefore, the applicability of RSM in capturing dynamic behavior and providing robust solutions for LWW may be limited.

### 4.2 Variability in real-world conditions

One of the challenges in performance evaluation of RSM-optimized treatment processes is the variability that can occur in real-world conditions. Factors such as changes in LWW compositions, full-scale reactor design, equipment, and ambient conditions can impact the actual performance of the optimized process, which may deviate from the predicted performance based on the RSM model. Furthermore, RSM requires a predefined range of input variable values for experimentation, which may not fully represent the wide variability of LWW characteristics and operating conditions [33]. This can result in suboptimal solutions if the optimal operating conditions lie outside the experimental range [11]. In addition, capturing and understanding the process complexity and interactions can be a challenge in performance evaluation of RSM-optimized treatment processes in the actual operational environment and may require further modeling techniques or experimental investigations [169]. Therefore, accounting for and mitigating the variability in real-world conditions can be a challenge in accurately evaluating the performance of RSM-optimized treatment processes.

### 4.3 Model validity and accuracy

The accuracy and validity of the RSM model used for process optimization and performance evaluation is crucial for obtaining reliable results [170]. The RSM model is typically developed based on obtained data, and the quality of the model depends on factors such as the experimental design, sample size, and statistical assumptions [171]. Ensuring that the RSM model is accurate and valid, and properly represents the underlying process, can be challenging and requires careful consideration of these factors.

### 4.4 Data availability and quality

The availability and quality of the data used for performance evaluation can also be a challenge. Sufficient and high-quality data are required for both developing the RSM model and evaluating the actual performance of the optimized process. However, collecting and managing data, especially in real-time or large-scale processes, can be challenging. Data quality issues, such as measurement errors, missing data, or inconsistencies, can also impact the reliability of performance evaluation results.

### 4.5 Robustness and generalization

Another challenge is assessing the robustness and generalization of the optimized treatment process. RSM models are typically developed based on a specific range of process parameter values and experimental conditions and may not always be robust or generalize well to different operating conditions or process variations [172]. Evaluating the performance of the optimized process under different conditions and assessing its robustness and generalization can be challenging and may require additional experiments or simulations.

### 4.6 Lack of consideration for techno-economic factors

Livestock wastewater treatment processes are not only influenced by technical factors, but also by economic considerations such as capital and operating costs, return on investment, and financial feasibility [7]. However, RSM may not explicitly consider these techno-economic factors in the optimization process. The lack of economic feasibility analysis during optimization may limit the applicability of the developed process for practical decision-making.

In summary, although RSM is a useful optimization technique for LWW, the constraints including simplified modeling assumptions, limited range of input variables, lack of consideration for process dynamics, reliance on experimental data, absence of techno-economic analysis, and limited flexibility for nonlinearities and interactions need to be considered.

## 5 Potential improvements in RSM application in livestock wastewater treatment

### 5.1 Incorporation of advanced modeling techniques

The RSM can be combined with advanced modeling techniques such as machine learning, artificial intelligence, and data-driven modeling approaches, to improve the accuracy and robustness of the optimization results [173]. These advanced techniques can capture more complex process behaviors, interactions, and nonlinearities, and provide more accurate predictions of the response variable [174,175]. This can enhance the effectiveness of RSM in optimizing LWW treatment technologies.

### 5.2 Dynamic RSM

Dynamic RSM involves incorporating the time-varying nature of LWW treatment processes into the optimization process. This can account for changing influent characteristics, temperature, and hydraulic loading rates and provide time-dependent optimal operating conditions [176]. Dynamic RSM can capture the dynamic behavior of LWW treatment processes and provide more reliable and robust optimization results applicable to real-world dynamic conditions.

### 5.3 Expansion of experimental design space

Expanding the experimental design space by considering a wider range of input variable values can improve the applicability and generalizability of RSM results to different scenarios or conditions [177]. This can involve conducting experiments under extreme or boundary conditions, as well as considering additional factors or variables that may impact the performance of LWW treatment processes. A more comprehensive experimental design can provide a more accurate representation of real-world conditions and improve the reliability of RSM optimization results.

### 5.4 Integration of multi-objective optimization

Livestock wastewater treatment processes often involve multiple conflicting objectives, such as cost minimization, environmental compliance, and social acceptability. Integrating multi-objective optimization techniques into RSM can enable simultaneous optimization of multiple objectives, considering their trade-offs [111,178]. This can provide a more comprehensive and practical approach to optimize LWW processes, considering multiple performance criteria and constraints.

### 5.5 Sensitivity analysis

Conducting a sensitivity analysis can provide insights into and improve the reliability of RSM optimization results by identifying the response variable(s) sensitivity to changes in input variables and quantifying the uncertainty in the model predictions [179]. This can help identify critical factors that significantly impact the performance of LWW treatment processes and to further understand robustness of the optimization results under uncertain conditions. Sensitivity analysis of input parameters on target response(s) can be assessed using Pareto analysis and relative contribution in the context of RSM [180,181]. In short, a sensitivity analysis can provide insights into the reliability and robustness of RSM-based optimization results and guide decision-making.

### 5.6 Integration of techno-economic analysis

Integrating techno-economic analysis into the RSM optimization process can ensure the economic viability and sustainability of the optimized treatment processes [182,183]. This can involve considering capital and operating costs, return on investment, and financial feasibility as additional optimization objectives or constraints. Techno-economic analysis can provide a more comprehensive assessment of the practical feasibility and economic benefits of the optimized LWW treatment processes and aid in making informed decisions.

### 5.7 Consideration of environmental and social factors

Livestock wastewater treatment processes are subject to various environmental and social regulations, such as discharge limits, odor control, and social acceptability [184,185]. Considering these factors explicitly in the RSM optimization process can ensure compliance with environmental and social requirements. This can involve incorporating environmental and social constraints, objectives, or penalties into the optimization model, and obtaining solutions that are not only technically optimal but also environmentally and socially sustainable.

### 5.8 Integration of real-time monitoring and control

Real-time monitoring and control technologies, such as process analyzers, sensors, and advanced process control systems, can enable continuous monitoring and adjustment of the treatment process in real-time [54,186]. Integrating real-time monitoring and control into the RSM optimization process can enable dynamic optimization and control of the treatment process based on changing conditions and provide more accurate and up-to-date performance evaluation results. Real-time monitoring and control can also facilitate the adaptive and responsive operation of LWW processes, improving their performance and sustainability.

## 6 Future research directions

**Fig. 4:**
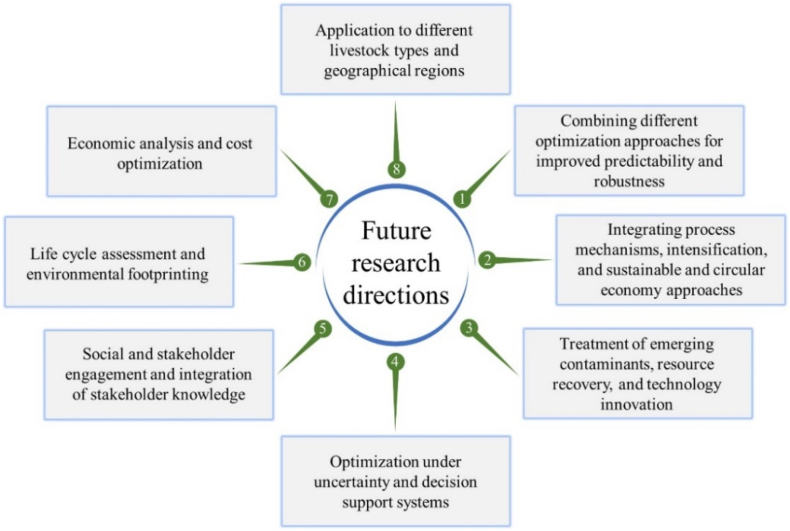
Potential areas of RSM application in LWW treatment.

Response surface methodology has shown great potential in designing ecofriendly, efficient, and sustainable LWW treatment processes. However, there are several future research directions that could further enhance the application of RSM in this field. A schematic of future research directions regarding RSM application in treating LWW is elucidated in Fig. 4.

The accuracy, robustness, and prediction ability of RSM can be further developed by combining it with other modeling techniques, such as gray relational analysis-based Taguchi design, artificial neural network, genetic algorithm models, and nonlinear programming [33,175,187]. Such integration could involve exploring new algorithms, models, and techniques that can help understand process dynamics, identify key variables, and elucidate interactive effects.

Research in RSM application could also extend to developing models that combine biological, chemical, and physical mechanisms to develop and optimize the integrated LWW treatment processes based on the underlying process kinetics and dynamics for efficient pollutant removal. Furthermore, the environmental, economic, and social aspects of the treatment processes, and optimizing the performance of the processes based on sustainability criteria, such as life cycle assessment, carbon footprint, and social impact assessment can be incorporated. This could also include studies related to the potential of circular economy approaches in LWW treatment processes.

Another potential area of application could be in discovering innovative technologies based on nanotechnology and advanced sensors to treat emerging contaminants (antibiotics, hormones, and microplastics) present in LWW and recover resources (nutrients, energy, and water) from livestock waste streams. This may entail integrating adsorption capacity, degradation kinetics, environmental fate, and recovery potential into the optimization models, as well as developing approaches to enhance the emerging contaminants removal and resource recovery efficiency from LWW.

Further studies can also be conducted by incorporating uncertainty quantification and risk analysis into RSM optimization models for LWW treatment processes. This could reflect uncertainties in input variables, model parameters, and measurement errors [188,189] and ultimately help develop strategies to optimize the performance of the LWW treatment processes under uncertain conditions. In addition, this could center on the advancement of developing robust optimization approaches that can provide reliable results even in the presence of uncertainties. Moreover, incorporating decision support systems based on real-time monitoring and control technologies, process simulation models, and economic analysis tools could be considered in the RSM-optimized LWW treatment process. Such an optimized and integrated decision support system will help LWW treatment plant operators and decision-makers to make informed decisions on optimal operating conditions, process adjustments, and resource allocation in the treatment processes and might be used in solving complex and dynamic issues related to control and automation of the LWW treatment plants [190]. This could also facilitate the adaptive and responsive operation of treatment processes based on changing conditions and requirements.

Considering the social, cultural, and stakeholder aspects of LWW processes in RSM optimization models is likely to be another area of future research. This might include social acceptability, stakeholder preferences, and community engagement into the optimization process to ensure that the optimized treatment processes are socially acceptable and aligned with the needs and expectations of the stakeholders. Furthermore, local and indigenous knowledge, expertise, and perspectives of stakeholders, such as farmers, regulators, and communities, could be incorporated into RSM optimization models. This could involve participatory approaches, stakeholder engagement processes, and knowledge co-creation methods that enable stakeholders to actively contribute to the optimization process, share their knowledge and experiences, and co-develop optimal solutions that are culturally, socially, and environmentally appropriate.

Further application of RSM in LWW treatment processes might be done for different types of livestock waste streams, such as poultry, swine, and dairy, and in different geographical regions with varying environmental, regulatory, and socio-economic conditions. This could employ adapting and customizing RSM optimization models to specific livestock types and regions, considering variations in wastewater characteristics, treatment technologies, and regulatory requirements. Exploring the applicability of RSM in developing countries and regions with limited resources and infrastructure for LWW and identifying cost-effective and sustainable treatment solutions can also be taken into account.

Moreover, life cycle assessment (LCA) and environmental foot printing approaches into RSM optimization models might be incorporated to evaluate the environmental impacts of LWW treatment processes holistically [191,192]. This will help understand the environmental impacts of the entire life cycle of the treatment processes, including construction, operation, maintenance, and disposal phases, and process optimization based on their overall environmental performance.

The integration of economic analysis and cost optimization approaches into RSM optimization models can also be considered for LWW processes. This could involve considering the economic feasibility, cost-effectiveness, and affordability of treatment technologies, and optimizing the processes based on economic criteria, such as capital and operational costs, return on investment, and cost-benefit analysis [193,194]. Such studies will help implement optimized treatment technologies in livestock farming operations in the most economical way.

Overall, the research directions discussed above would support the development of eco-friendly, efficient, and effective LWW treatment technologies that can mitigate environmental pollution, protect public health, and support sustainable livestock farming practices.

## 7 Conclusion

High pollutant-laden livestock waste streams pose a significant detrimental impact on environmental and human health. For the past several decades, researchers around the globe have therefore developed and tested several biological, physical, and chemical LWW treatment processes that demonstrate better performance when optimized. Among the different optimization techniques, RSM is one of the most widely adopted approaches owing to its accurate and robust prediction potential. Optimization of LWW treatment processes using RSM can provide valuable insights into the interactions between different process variables, their effects on treatment performance, and optimal operating conditions for achieving desired treatment outcomes. Moreover, RSM enables adaptive and responsive operation of treatment processes by providing a framework to monitor and adjust operating parameters based on changing conditions and requirements. Such flexibility ensures that treatment systems remain effective and efficient over time and empowers operators and decision-makers to make informed choices and meet regulatory standards. Nonetheless, RSM is most suitable for optimizing four to five operational parameters. The inclusion of more than five parameters leads to an increase in the number of experiments. Integrating machine learning, artificial intelligence, process simulation models, economic analysis tools, and real-time monitoring into RSM can help further improve its predictive capability. Overall, RSM-optimized LWW treatment processes could lead to improved pollutant removal efficiencies, enhanced sustainability, better alignment with stakeholder needs and expectations, and thus contributing to the development of more efficacious, cost-effective, and eco-friendly LWW treatment technologies.

## Data availability statement

This is a systematic review; therefore, all the data are included in the manuscript.

## CRediT authorship contribution statement

**Arif Reza:** Conceptualization, Data curation, Methodology, Visualization, Writing – original draft, Writing – review & editing. **Lide Chen:** Conceptualization, Funding acquisition, Methodology, Project administration, Supervision. **Xinwei Mao:** Resources, Writing – review & editing.

## Declaration of competing interest

The authors declare that they have no known competing financial interests or personal relationships that could have appeared to influence the work reported in this paper.
